# Estimated Prevalence of Nonverbal Learning Disability Among North American Children and Adolescents

**DOI:** 10.1001/jamanetworkopen.2020.2551

**Published:** 2020-04-10

**Authors:** Amy E. Margolis, Jessica Broitman, John M. Davis, Lindsay Alexander, Ava Hamilton, Zhijie Liao, Sarah Banker, Lauren Thomas, Bruce Ramphal, Giovanni A. Salum, Kathleen Merikangas, Jeff Goldsmith, Tomas Paus, Katherine Keyes, Michael P. Milham

**Affiliations:** 1 Division of Child and Adolescent Psychiatry, Department of Psychiatry, Vagelos College of Physicians & Surgeons, Columbia University Irving Medical Center, New York, New York; 2 International Control Mastery Therapy Center, Berkeley, California; 3 Department of Educational Psychology, California State University, Hayward; 4 Center for the Developing Brain, Child Mind Institute, New York, New York; 5 Department of Epidemiology, Mailman School of Public Health, Columbia University Irving Medical Center, New York, New York; 6 Bloorview Research Institute, Holland Bloorview Kids Rehabilitation Hospital, Toronto, Ontario, Canada; 7 Department of Psychology, University of Toronto, Toronto, Ontario, Canada; 8 Department of Psychiatry, Universidade Federal do Rio Grande do Sul, Porto Alegre, Brazil; 9 Section on Negative Affect and Social Processes, Hospital de Clínicas de Porto Alegre, Porto Alegre, Brazil; 10 Intramural Research Program, National Institute of Mental Health, Bethesda, Maryland; 11 Department of Biostatistics, Mailman School of Public Health, Columbia University Irving Medical Center, New York, New York; 12 Bloorview Research Institute, Holland Bloorview Kids Rehabilitation Hospital, Toronto, Ontario, Canada; 13 Department of Psychology, University of Toronto, Toronto, Ontario, Canada; 14 Department of Psychiatry, University of Toronto, Toronto, Ontario, Canada; 15 Center for Biomedical Imaging and Neuromodulation, Nathan S. Kline Institute for Psychiatric Research, Orangeburg, New York

## Abstract

**Importance:**

Nonverbal learning disability (NVLD) is a neurodevelopmental disorder characterized by deficits in visual-spatial processing but not in reading or verbal ability; in addition, problems in math calculation, visual executive functioning, fine-motor skills, and social skills are often present. To our knowledge, there are no population-based estimates of the prevalence of NVLD in community samples.

**Objective:**

To estimate the prevalence of the NVLD cognitive profile in 3 independent samples of children and adolescents from studies centered around brain imaging in the US and Canada.

**Design, Setting, and Participants:**

This cross-sectional study used data from 2 samples recruited from the community and overselected for children with psychiatric disorders (Healthy Brain Network [HBN], January 1, 2015, to December 31, 2019, and Nathan Kline Institute–Rockland Sample [NKI], January 1, 2011, to December 31, 2018) and 1 community-ascertained population sample (Saguenay Youth Study [SYS], January 1, 2003, to December 31, 2012) overselected for active maternal smoking during pregnancy.

**Main Outcomes and Measures:**

Prevalence of NVLD. Criteria for NVLD were based on clinical records of deficits in visual-spatial reasoning and impairment in 2 of 4 domains of function (fine-motor skills, math calculation, visual executive functioning, and social skills). Sample weighting procedures adjusted for demographic differences in sample frequencies compared with underlying target populations. Inflation factor weights accounted for overrepresentation of psychiatric disorders (HBN and NKI samples).

**Results:**

Across 3 independent samples, the prevalence of NVLD was estimated among 2596 children and adolescents aged 6 to 19 years (mean [SD] age, 12.5 [3.4] years; 1449 male [55.8%]). After sample and inflation weights were applied, the prevalence of NVLD was 2.78% (95% CI, 2.03%-3.52%) in the HBN sample and 3.9% (95% CI, 1.96%-5.78%) in the NKI sample. In the SYS sample, the prevalence of NVLD was 3.10% (95% CI, 1.93%-4.27%) after applying the sample weight. Across samples and estimation strategies, the population prevalence of NVLD was estimated to range from 3% to 4%. When applied to the US population younger than 18 years, 2.2 million to 2.9 million children and adolescents were estimated to have NVLD.

**Conclusions and Relevance:**

The findings suggest that the prevalence of NVLD in children and adolescents may be 3% to 4%. Given that few youths are diagnosed with NVLD and receive treatment, increased awareness, identification of the underlying neurobiological mechanisms, and development and testing interventions for the disorder are needed.

## Key Points

**Question:**

What is the prevalence of nonverbal learning disability among children and adolescents in North America?

**Findings:**

In this secondary analysis of a cross-sectional study of 2596 children and adolescents in North America, the population prevalence of nonverbal learning disability ranged from 3% to 4%.

**Meaning:**

This study showed that 2.2 million to 2.9 million children and adolescents may have nonverbal learning disability; further studies appear to be needed to develop and test interventions for treatment of this disorder.

## Teaser

This secondary analysis of a cross-sectional study estimates the prevalence of the nonverbal learning disability cognitive profile in children and adolescents from studies centered around brain imaging in the US and Canada.

## Introduction

Clinical interest in nonverbal learning disability (NVLD) dates back to 1967, when the disorder was first described.^1^ Children with NVLD were identified as having deficits in visual-spatial abilities instead of more commonly studied language abilities. Subsequently, practitioners and researchers^2,3,4,5,6,7^ have continued to describe a subset of children with visual-spatial deficits as well as impairments in social skills and/or school functioning who do not demonstrate repetitive interests or motor acts, distinguishing them from children on the autism spectrum. Although NVLD is currently not included in the *Diagnostic and Statistical Manual of Mental Disorders* (Fifth Edition) (*DSM-5*)^8^ and there has been some lack of consensus on the criteria for the disorder, previous studies^9,10,11,12^ suggest convergence on a definition. Reviews of the empirical literature^11,12^ suggest that NVLD is best described by deficits in visual-spatial ability or a discrepancy between visual-spatial and verbal ability accompanied by problems in math calculation but not basic reading or spelling skills. In addition, problems in visual-spatial memory, attention, visual executive functioning, fine-motor skills, and social skills are often present. This definition has been operationalized in previous empirical studies of the neuropsychological profile,^13^ brain structure,^14^ and function^15,16^ of children with NVLD (Table 1).

**Table 1.** Criteria for Nonverbal Learning Disability Diagnosis

Domain	Evidence^a^
Step 1	
Visual-spatial deficit	Performance ≤16th percentile on visual-spatial tests or significant discrepancy between verbal and spatial abilities (eg, VIQ-PIQ ≥15 points)
Intact single-word reading abilities	Performance >16th percentile
Step 2	
Social skills	Deficit in at least 2 of 4 areas: performance ≤16th percentile
Visual executive functioning	
Math calculation	
Motor skills	
Step 3: absence of ASD features	ASD features ruled out by survey or by study exclusion criteria

Abbreviations: ASD, autism spectrum disorder; PIQ, performance IQ; VIQ, verbal IQ.

^a^ eTable 1 in the Supplement gives a complete list of measures used in each data set to determine eligibility for nonverbal learning disability.

Although NVLD has been discussed for more than 50 years, few studies have estimated its prevalence, likely because of a lack of consensus about a definition or a lack of awareness. Extant prevalence studies^17,18,19,20^ were conducted with small samples of children referred for assessment of learning problems who do not generalize to the broader child population. Ozols and Rourke^17^ reported that 10% of a sample of children diagnosed with a learning disability (LD) met study-defined criteria for NVLD. However, a later report^6^ suggested that the prevalence in a sample of children with LD could be less than 1%. Other researchers estimated the prevalence of NVLD among children with LD to be as high as 25%^18^ or as low as 3%.^19^ Most recently, it was reported that 2% of children with LD met the criteria for NVLD.^20^

Estimates of NVLD range from 1% to 25% of children with known LD, with most estimates being toward the lower end.^17,18,19,20^ Given that LD is commonly estimated to affect between 5% and 15% of the population,^8^ the prevalence of NVLD could be expected to range from 0.05% to 3.75%. The wide range of these estimates, spanning 2 orders of magnitudes, necessitates additional research into the prevalence of NVLD. Ideally, such an examination would be performed in a population sample, not a sample focused on LD alone, which has been the common method to date. However, the appropriate phenotyping is rarely included in population samples because it is time intensive and expensive to acquire.

To address this gap in knowledge, we leveraged the inclusion of sufficient phenotypic data in 3 large-scale samples from studies centered around brain imaging to ascertain an updated estimate of NVLD prevalence. These samples included the Healthy Brain Network (HBN) sample, a community–self-referred sample focused on mental health and learning disorders^21^; the Nathan Kline Institute–Rockland Sample (NKI), a community-ascertained sample focused on normative and abnormal brain development^22^; and the Saguenay Youth Study (SYS) sample, a community-ascertained population sample focused on brain and cardiometabolic health in adolescents who differed in their exposure to maternal cigarette smoking during pregnancy.^23^

Specifically, the demographic characteristics of community-based samples may not be representative of the general population from which the samples were drawn, and thus sample weighting was used to better approximate prevalence in these target populations. We expected that HBN and NKI would likely overrepresent psychiatric disorders even after accounting for differences in demographic characteristics, particularly the HBN because of self-referral basis of study participation. We attempted to account for lack of external validity to the target population by generating a disorder-based inflation weight, a correction strategy that estimates inflation of prevalence in the sample based on the estimated national prevalence of common disorders of childhood. We expected that rates of disorders characterized by a high degree of externalizing or disruptive behaviors (eg, attention-deficit/hyperactivity disorder [ADHD] and autism spectrum disorder [ASD]) would be more inflated than rates of internalizing disorders (eg, anxiety and depression). We then used SYS, a population sample ascertained by exposure to maternal cigarette smoking during pregnancy and not by child behavior or psychiatric symptoms to test whether these sample- and disorder-adjusted prevalence estimates of NVLD in the psychiatrically overrepresented samples were consistent with population rates. Last, we evaluated the rates of *DSM-5* diagnoses ascribed to children identified as meeting the criteria for NVLD in the HBN and NKI samples.

## Methods

### Participants

This cross-sectional study used data from 3 large-scale neuroimaging samples overselected for active maternal smoking during pregnancy.(HBN [January 1, 2015, to December 31, 2019], NKI [January 1, 2011, to December 31, 2018], and SYS [January 1, 2003, to December 31, 2012]) to evaluate the prevalence of NVLD. The HBN study was approved by the Chesapeake Institutional Review Board. Before conducting the research, written informed consent was obtained from participants 18 years or older. For participants younger than 18 years, written informed consent was obtained from their legal guardians and written informed assent was obtained from the participant. The NKI study was approved by the institutional review board at the Nathan Kline Institute and at Montclair State University. Written informed consent was obtained for all study participants. Written informed consent and assent were also obtained from minor or child participants and their legal guardians. The SYS study was approved by the Chicoutimi Hospital Research Ethic Committee. Written informed consent and assent were obtained from the parents and adolescents, respectively. The current secondary analysis of deidentified data was deemed not to meet the definition of human subjects research by the institutional review board of the New York State Psychiatric Institute; therefore, participant consent or assent was not obtained for this analysis. This study followed the Strengthening the Reporting of Observational Studies in Epidemiology (STROBE) reporting guideline.

 Records were included if they contained complete data on the demographic variables used to create the weights. In addition, samples were restricted to records that included the required neuropsychological data, which in turn dictated the age range of each sample because specific tests were administered at particular ages. Records with Autism Spectrum Screening Questionnaire scores of 19 or higher were excluded from the HBN sample to increase the comparability among the samples. Secondary analyses were conducted to confirm that this decision did not substantively influence the estimate of NVLD prevalence in the HBN sample. In the HBN sample, 1283 participants had available data on sex and both race and ethnicity. In the NKI sample, 309 participants had available data on race and sex. In the SYS sample, 1004 participants had complete data on paternal educational level. Because the SYS sample has a known founder effect, race and ethnicity were not used to create sample weights.

#### HBN Sample

The HBN sample is currently enrolling a sample of 10 000 children and adolescents (aged 5-21 years) who reside in the New York City area of New York^21^; a total sample size of 1283 children and adolescents aged 6 to 19 years were included in the present analysis. The HBN adopts a self-referred community recruitment model, recruiting participants on the basis of perceived clinical concern (mental health or learning). This strategy promotes the inclusion of a high proportion of youths with behavioral or emotional problems, thereby increasing the probability of clinically significant conditions that could form the basis for nested case-control studies.

#### NKI Sample

The NKI sample is a large-scale community sample of participants across the life span and is intended to be a phenotypically rich neuroimaging sample that consists of data obtained from individuals aged 6 and 85 years.^22^ A total sample size of 309 children and adolescents aged 6 to 17 years was used for the present analysis. Zip code–based recruitment (eg, advertisement flyer mailings and posting of materials in local shops and meeting places) and enrollment efforts were used to avoid overrepresentation of any portion of the community and to ensure geographic diversity throughout Rockland County. All individuals included in the sample underwent semistructured diagnostic psychiatric interviews and completed psychiatric, cognitive, and behavioral assessments.

#### SYS Sample

The SYS sample is a community-ascertained sample of Canadian individuals living in the Saguenay Lac Saint-Jean region of Quebec, Canada.^23^ This 2-generational study^23^ of adolescents and their parents aims to investigate trajectories of cardiometabolic and brain health. A sample of 1004 adolescents aged 12 to 19 years was used in the current analyses. All individuals completed demographic surveys, neuropsychological and cardiometabolic assessments, and brain and body imaging.^23^ Half of the sample was recruited for active maternal smoking during pregnancy, whereas the other half was matched on maternal demographic characteristics, resulting in a lower mean maternal educational level across the sample.

### Measures Used to Identify NVLD in Each Sample

Identification of NVLD was established in accord with recent reviews of the definition^11,12^ and definitions used in prior empirical studies (Table 1).^13,14,15,24^ Children were included in the NVLD group following a 3-step process. First, they had to have visual-spatial deficits (performance ≤16th percentile) as measured by a Wechsler Intelligence Scale or a discrepancy between verbal and visual-spatial abilities of 15 points or more and intact single-word reading abilities (or a proxy thereof). Second, they had to have deficits in at least 2 domains: fine motor skills, math calculation, visual executive functioning, or social skills. Third, they could not have evidence of ASD. Specific measures for each sample are given in eTable 1 in the Supplement.

### Sample Weighting

We used statistical raking to generate sample weights for each of 3 samples. Specifically, we obtained data on the total number of children in the same age range of the children in the analyzed data in each region: Metropolitan New York (n = 1 340 375), Rockland County (n = 59 807), and Saguenay (n = 7533). eTable 2 in the Supplement describes the population data that were available for the 3 areas and the demographic characteristics that were used to create the sample weights based on these population data. In statistical raking procedures, distributions of the sample that are underrepresented given their prevalence in the target population are given higher weights than characteristics of the sample that are overrepresented given their prevalence in the target population. eTable 2 in the Supplement provides an overview of the distributions of demographic characteristics in the populations (preweighted and postweighted samples). After raking, study characteristics were weighted to reflect their distributions in the general population within a caliper of error.^25,26^ To control for outliers in the sample weight, we also created a sensitivity weight for which we trimmed the top 1% of the created sample weights.

### Inflation Factor Weighting

The HBN and NKI samples were self-referred, and thus prevalence estimates might not be generalizable to the general population; higher frequencies of disorders were expected in the HBN and NKI samples than in the general population. Furthermore, we expected that disorders characterized by relative high rates of externalizing or disruptive disorders (eg, ADHD and ASD) would be more inflated than rates of internalizing disorders (eg, anxiety and depression). To adjust for this sampling bias, we created disorder-specific inflation factor weights by dividing the sensitivity-weighted rate of each common childhood disorder in each community sample by the known prevalence of the disorder. Rates of depression, anxiety, and ADHD were obtained from the National Institutes of Health website,^27^ rates of specific learning disorders from the National Center for Education Statistics website,^28^ and rates of ASD from the Centers for Disease Control and Prevention website.^29^ Details on which data were selected for estimating population rates is presented in the eMethods in the Supplement.

### Statistical Analysis

We estimated the prevalence of NVLD in each sample before and after sample weighting. To adjust for any sampling bias in the HBN and NKI samples, we adjusted the estimated NVLD rate with the sample-specific ADHD inflation factor weight. We selected ADHD as the inflation factor because (1) children with NVLD often have overt attention problems, suggesting that they may be overrepresented in the samples at rates similar to the overrepresentation of ADHD in the samples, and (2) children with ADHD were present in all samples (as opposed to ASD, which had a comparable inflation factor in the HBN sample). We then compared our prevalence estimates in the HBN and NKI samples with the estimated prevalence in the SYS sample, a community-ascertained sample. The 95% CIs for all estimated rates of NVLD were obtained through a resampling procedure that accounted for uncertainty in raking and the effect of trimming to obtain sensitivity weights. To better understand the way that individuals with NVLD are currently represented in clinics and in large data sets, the frequency of *DSM-5* diagnoses among children who met criteria for NVLD in the HBN and NKI samples was calculated. Data analysis was performed using R, version 2.6.1 (R Foundation for Statistical Analysis) and SAS, version 9.4 (SAS Institute).

## Results

### Samples

Across 3 independent samples, the prevalence was estimated in 2596 children and adolescents aged 6 to 19 years (mean [SD] age, 12.5 [3.4] years; 1449 male [55.8%]). Demographic characteristics of each sample are presented in eTables 3 through 5 in the Supplement. The unweighted HBN and NKI samples were compared with population totals derived from the American Community Survey (n = 59 807) (eTable 3 and eTable 4 in the Supplement) for children aged 3 to 18 years; the SYS sample was compared with the Canadian Census in Saguenay (n = 7533) (eTable 5 in the Supplement). After weighting, no differences were found among the weighted HBN, NKI, or SYS samples and their respective comparison population in the distributions of age, race/ethnicity, sex, or income for the HBN and NKI samples and for age, parental educational level, or parental income for the SYS sample. Demographic differences in sample frequencies compared with the underlying target populations are presented in the eAppendix in the Supplement.

### Inflation Factor Weight in the HBN and NKI Samples

**Figure 1.**
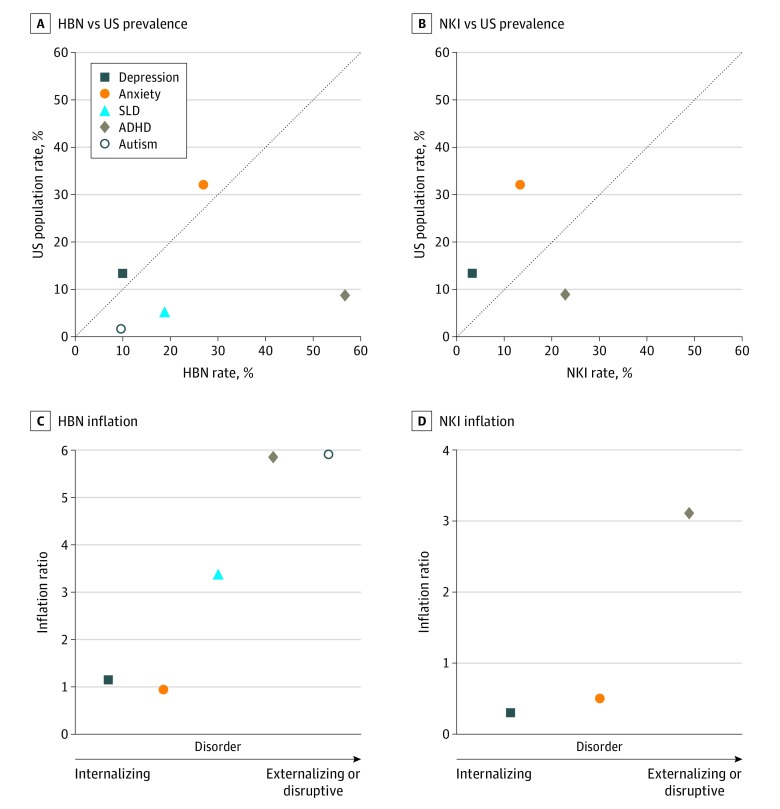
Prevalence of Psychiatric Disorders in Healthy Brain Network (HBN) and Nathan Kline Institute (NKI) Samples vs Prevalence in the US A and B, Points closer to the identity line represent the convergence of sample and population estimates. C and D, The extent to which a disorder is overrepresented varies with the extent to which the disorder lies on a spectrum from internalizing to externalizing or disruptive behaviors. ADHD indicates attention-deficit/hyperactivity disorder; SLD, specific learning disorder.

Externalizing or disruptive behavior disorders (ASD and ADHD) were more overrepresented than internalizing disorders in the HBN and NKI samples; inflation factors ranged from 3.1 to 6.2 for ADHD and ASD and 0.3 to 1.0 for depression and anxiety (Figure 1 and eTable 6 in the Supplement). Visual inspection showed that the degree of externalizing or disruptive behavior characteristics was associated with increased inflation compared with the estimated population rate for each disorder (Figure 1). The ADHD inflation rates were 6.2% in the HBN sample and 3.1% in the HBN sample.

### Prevalence of NVLD

In the HBN sample, the prevalence of NVLD was 10.68% (n = 137) before and 17.13% (95% CI, 12.54%-21.73%) after applying the sensitivity sample weights (Table 2). Rates were similar when records with Autism Spectrum Screening Questionnaire scores of 19 or higher were not excluded (eTable 7 in the Supplement). In the NKI sample, the prevalence of NVLD was 9.71% before and 11.81% (95% CI, 5.98%-17.64%) after applying the sensitivity sample weights (Table 2). After the respective inflation weight for ADHD was applied to each sample, the prevalence of NVLD was 2.78% (95% CI, 2.03%-3.52%) in the HBN sample and 3.87% (95% CI, 1.96%-5.78%) in the NKI sample. In the SYS sample, the prevalence of NVLD was 2.89% before and 3.10% after (95% CI, 1.93%-4.27%) applying the sensitivity sample weight (Table 2). Across samples and estimation strategies, the population prevalence of NVLD was estimated to range from 3% to 4%. When we applied these strategies to the US population younger than 18 years, we estimated that 2.2 million to 2.9 million children and adolescents in the US may have NVLD. The demographic characteristics of children who met criteria for NVLD are presented in the eAppendix in the Supplement.

**Table 2.** Prevalence of NVLD in Each Sample

Sample	Met criteria for NVLD, %			Adjusted rate of NVLD among population^b^
	Unweighted	Weighted	Sensitivity weighted (95% CI)^a^	
HBN	10.68	22.11	17.13 (12.54-21.73)	2.78 (2.03-3.52)
NKI	9.71	12.23	11.81 (5.98-17.64)	3.87 (1.96-5.78)
SYS	2.89	3.10	3.10 (1.93-4.27)	NA

Abbreviations: ADHD, attention-deficit/hyperactivity disorder; HBN, Healthy Brain Network; NA, not applicable; NKI, Nathan Kline Institute–Rockland Sample; NVLD, nonverbal learning disability; SYS, Saguenay Youth Study.

^a^ With a 1% trim.

^b^ Sensitivity-weighted NVLD rate divided by weighted ADHD inflation rate.

### Rates of *DSM-5* Diagnoses Among Youths Who Met the NVLD Criteria

**Figure 2.**
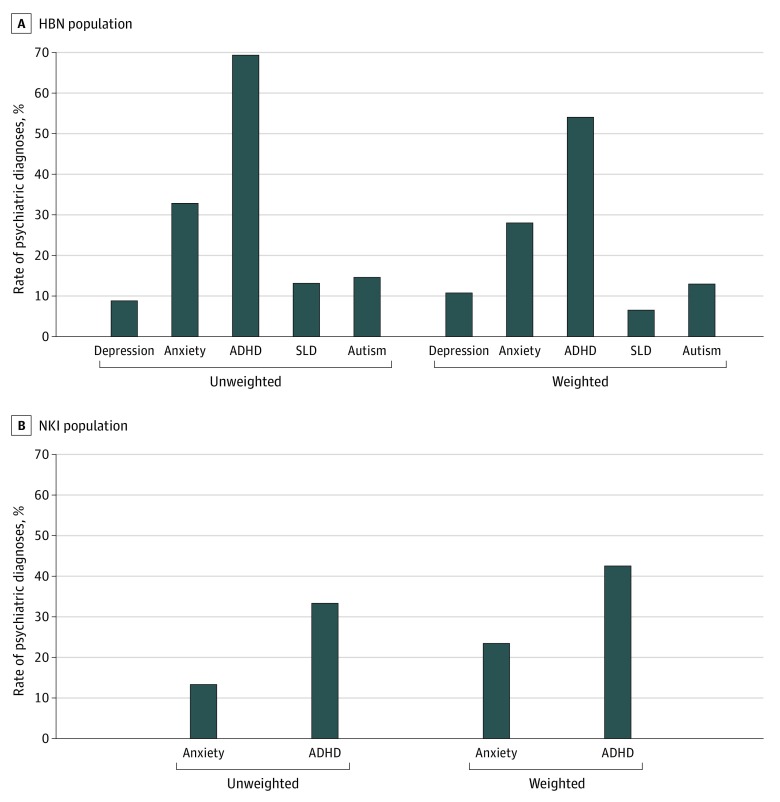
Unweighted and Sample Weighted Rates of Psychiatric Diagnoses Among Participants With Nonverbal Learning Disorder in Healthy Brain Network (HBN) and Nathan Kline Institute (NKI) Samples ADHD indicates attention-deficit/hyperactivity disorder; SLD, specific learning disorder.

 In the HBN sample, the most common diagnosis received by children who met criteria for NVLD was ADHD (95 [69.3%]) followed by anxiety disorder (45 [32.9%]) and specific learning disorder (18 [13.1%]) (Figure 2). Similarly, in the NKI sample the most common diagnosis received by children who met criteria for NVLD was also ADHD (10 [33.3%]) followed by anxiety disorder (4 [13.3%]); Specific learning disorder was not clinically assessed in this sample. Ten youth (7.3%) youth in the HBN sample and 15 (50.0%) in the NKI sample did not meet criteria for any diagnosis.

## Discussion

The current study leveraged phenotypic data from 3 large-scale data sets centered around brain imaging to estimate the prevalence of NVLD. To our knowledge, this was the first study to estimate NVLD in large community-based samples. Prior estimates^6,17,18,19,20^ in samples of children with LD were reported to be 1% to 25%, suggesting a 0.05% to 3.75% rate in the general population. We estimated the prevalence of NVLD to range from 2.8% to 3.9% in 2 psychiatrically weighted cohorts. The prevalence estimate of NVLD in a community-ascertained sample was 3.1%. Such high convergence across data sets from 2 countries, all using slightly different measurement tools, suggests that the estimate is robust.

The prevalence of NVLD in the HBN and NKI samples increased after sample weights adjusted for demographic characteristics. This finding was surprising because we hypothesized that NVLD would be overrepresented in the samples given self-selection into studies of individuals with psychiatric problems. In the HBN sample, sample weights adjusted for underrepresentation of minority groups and low-income individuals. In the NKI sample, weights adjusted for underrepresentation of low-income individuals. In both samples, applying sample weights yielded increased NVLD prevalence. Our finding suggests that NVLD does not occur only in European, white individuals with high socioeconomic status.

Applying inflation factor weights to our sample aligned the NVLD estimate with rates of known disorders in the population. Clinical observation suggested that the behavioral features of ADHD would most closely mimic those of NVLD. ADHD presents similarly to NVLD in terms of symptoms that generally prompt parents to seek evaluation for their children (eg, difficulty in school or social situations and missing instructions). Thus, we hypothesized that inflation of ADHD would best estimate the inflation of NVLD in these clinically ascertained samples. Validating the a priori decision to use ADHD as the disorder by which to determine inflation rate, the most common diagnosis received among those identified as meeting criteria for NVLD in the HBN and NKI samples was ADHD. Of note, on assessment, NVLD is distinct from ADHD in that it is characterized by deficits in visual-spatial ability that underlie overt behaviors that overlap with frequently detected problems in children with ADHD. Examining the range of inflation weights in the HBN and NKI data sets revealed a pattern of increasing overrepresentation of disorders with increasing degrees of externalizing or disruptive behaviors. This finding suggests that increased awareness and knowledge about the signs of internalizing (low inflation weight) disorders may help pediatricians and parents become aware of these problems sooner, potentially leading to earlier treatment for children.

More generally, these results underscore the potential for clinical and community-based samples to provide information on population prevalence and associations when the desired target population is explicit. Psychiatric data are increasingly accumulated and available on general population and community-based samples compared with samples that are ascertained based on individuals who present for psychiatric care, increasing the potential for estimating population burden of psychiatric diagnoses. Although such samples provide information on clinical characteristics, the results may not be generalizable to nonclinical or general populations. For example, LeWinn et al^30^ found that in large community-ascertained samples of children, prevalence estimates and associations between age-related changes in brain structure and function changed substantially when data were weighted to be representative of the general population. Another study^31^ of volunteers and convenience methods lacked generalizability to broader populations. However, epidemiologic methods are rapidly developing to implement high-quality weighting algorithms, allowing for greater transparency and utility of clinical and community-based data.^32,33^

Identifying individuals who meet criteria for NVLD may have a significant and positive influence on their mental health. Between 7% and 50% of individuals who met criteria for NVLD obtained no diagnosis in HBN or NKI samples, indicating the importance of this diagnosis to obtaining treatment through increased access to mental health services for children whose conditions would be otherwise undiagnosed. The first and second most common diagnoses received by those identified as meeting criteria for NVLD were ADHD and anxiety disorder, respectively, which is consistent with clinical observations that individuals with NVLD often experience attention and anxiety problems.^10^ Such findings also suggest that these disorders may represent common comorbidities with NVLD. It is likely that the impairment experienced by individuals with NVLD differs from that in those with more typical anxiety or ADHD because the impairment in NVLD is thought to derive form underlying spatial deficits.^16^ Future research should begin to detail how spatial deficits manifest as functional impairments.

Although NVLD is not identified in *DSM-5*, the *International Statistical Classification of Diseases and Related Health Problems, Tenth Revision (ICD-10)* provides a code for individuals with visuospatial deficit (R41.842), which may capture NVLD. Future studies could investigate the presentation and cognitive profiles of those diagnosed with visuospatial deficit (*ICD-10* code R41.842) to examine trends in the developmental course and associated features of the disorder. Increased recognition of the unique characteristics of NVLD and inclusion of the diagnosis in future editions of the *DSM* may lead to increased development and deployment of specific NVLD-related interventions. In schools, increased awareness of the challenges that individuals with NVLD face may lead to increased accommodations and support.

### Limitations

This study has limitations. We estimated the prevalence of NVLD in 2 data sets in which the sampling strategy led to overrepresentation of psychiatric disorders. However, we applied sample weights and inflation factor weights to better approximate the rate in the general population. The consistency across samples with different recruitment methods provides a replication and validation of the rates obtained in any 1 sample. Future studies should estimate the prevalence of NVLD in community-ascertained samples that include younger children and older adults. Furthermore, the validation sample was obtained in Canada, and the other samples were from the Northeast US; thus, divergences associated with geographic or cultural contexts are possible. However, estimates were highly comparable across samples, pointing to the robustness of our findings. Visuomotor skill is a key component of the visual-spatial deficits that characterize NVLD.^34,35^ However, no direct measures of visuomotor skills were available in the data sets used in this study; future work would benefit from inclusion of a more comprehensive assessment of visual-spatial skills that would make detailed assessments of visual-spatial deficits possible. Although we did not have measures of gross motor skills, we believe that the estimates of NVLD were not confounded by developmental coordination disorder because only 61% of the those who met criteria for NVLD had fine-motor skill deficits. In addition, this study estimated prevalence from 3 existing samples, which differed with respect to the specific tests used to identify NVLD. Although the convergence of the findings from the different samples supports the robustness of the observed prevalence rates, future data collection would benefit from the establishment and use of standardized measures for detecting NVLD.

## Conclusions

We estimated that the prevalence of NVLD may be 3% to 4% in the general population, representing a substantial portion of children and adolescents. These findings were similar in a community-ascertained sample. At a national level, disorders with rates of 1% to 2%, such as ASD and schizophrenia, are associated with a significant public health burden. The current work suggests the need for inclusion of this diagnosis in *DSM* nosology, for future research on the neurobiological basis of NVLD, and for work aimed at developing intervention methods.

## Notes

Supplement. **eTable 1.** Specific Measures Available in Each Data Set **eTable 2.** Population and Sample Characteristics Used for Creating Sampling Weights Across 3 Neuroimaging Samples **eTable 3.** HBN and NYC* Populations and Variables Used to Create a Sample Weight **eTable 4.** NKI and Rockland* Populations and Variables Used to Create a Sample Weight **eTable 5.** SYS and Saguenay Populations and Variables Used to Create a Sample Weight **eTable 6.** Inflation Rate by Specific Disorder **eTable 7.** Prevalence of NVLD in HBN Without Excluding ASSQ≥19 **eMethods.** Supplemental Methods **eAppendix.** Supplemental Results
