# Supplementary material for: Estimated Prevalence of Nonverbal Learning Disability Among North American Children and Adolescents
Source: JAMA Netw Open. 2020 Apr 10;3(4):e202551. doi: 10.1001/jamanetworkopen.2020.2551 (PMC7148441; doi:10.1001/jamanetworkopen.2020.2551)
Supplement: Supplement. — eTable 1. Specific Measures Available in Each Data Set eTable 2. Population and Sample Characteristics Used for Creating Sampling Weights Across 3 Neuroimaging Samples eTable 3. HBN and NYC* Populations and Variables Used to Create a Sample Weight eTable 4. NKI and Rockland* Populations and Variables Used to Create a Sample Weight eTable 5. SYS and Saguenay Populations and Variables Used to Create a Sample Weight eTable 6. Inflation Rate by Specific Disorder eTable 7. Prevalence of NVLD in HBN Without Excluding ASSQ≥19 eMethods. Supplemental Methods eAppendix. Supplemental Results [file jamanetwopen-3-e202551-s001.pdf]

Supplementary Online Content

Margolis AE, Broitman J, Davis JM, et al. Estimated prevalence of nonverbal learning disability among North American children and adolescents. *JAMA Netw Open*. 2020;3(4):e202551. doi:10.1001/jamanetworkopen.2020.2551

eTable 1. Specific Measures Available in Each Data Set

eTable 2. Population and Sample Characteristics Used for Creating Sampling Weights Across 3 Neuroimaging Samples

eTable 3. HBN and NYC* Populations and Variables Used to Create a Sample Weight

eTable 4. NKI and Rockland* Populations and Variables Used to Create a Sample Weight

eTable 5. SYS and Saguenay Populations and Variables Used to Create a Sample Weight

eTable 6. Inflation Rate by Specific Disorder

eTable 7. Prevalence of NVLD in HBN Without Excluding ASSQ ≥19

eMethods. Supplemental Methods

eAppendix. Supplemental Results

This supplementary material has been provided by the authors to give readers additional information about their work.

© 2020 Margolis AE et al. *JAMA Network Open.*

eTable 1. Specific Measures Available in Each Data Set

Domain							
	Step 1		Step 2				Step 3
Sample	Spatial Deficit	Word Reading	Social	EF	Math	Motor	ASD features
HBN	WISC/WASI/WAIS MR or BD ≤ 16th %ile OR WISC 5 VIQ-PIQ OR VIQ-FRI > 15 points OR WAIS/WASI VCI-PRI > 15	WIAT Word reading ≥16th%ile	CBCL Social Problems T ≥ 70	NIH Flanker or Card Sort ≤ 16th%ile	WIAT Numerical Operations ≤ 16th%ile	Grooved Pegboard ≤ 16th%ile	ASSQ <19
NKI	WASI MR or BD ≤ 16th %ile OR VIQ-PIQ > 15 points	WIAT Word reading ≥16th%ile	CBCL Social Problems ≥ 95th%ile	DKEFS Trail Making, Card Sort ≤16th%ile	WJ-III Calculation ≤16th%ile	Perdue Pegboard ≤16th%ile	ASD is exclusion for the study
SYS	WISC -III PIQ ≤ 16th%ile OR VIQ-PIQ > 15 points	WJ Spelling ≥16th%ile	Positive Youth Development Scale ≤ 16th%ile	Stroop Interference ≤16th%ile	WJ-III Calculation ≤16th%ile	Grooved Pegboard ≤ 16th%ile	ASD is exclusion for the study

*For HBN and NKI samples 16th%ile is based on standardized national norms. For SYS the 16th%ile is calculated within the sample itself.

© 2020 Margolis AE et al. *JAMA Network Open.*

eTable 2. Population and Sample Characteristics Used for Creating Sampling Weights Across 3 Neuroimaging Samples

	**Population characteristics available**	**Sample characteristics available**
HBN (N=1350)	*American Community Survey,2016:*<br>**Age**: Age groups (5 to 9, 10 to 14, 15 to 17)<br>**Sex**: Male or female<br>**Race/Ethnicity**: Hispanic, non-Hispanic White, Black, American Indian and Alaska Native, Asian, Native Hawaiian and Other Pacific Islander, other race, two or more races<br>**Socio Economic Status**: Above/below the poverty line, missing	**Age**: Single ages grouped into three age groups (5 to 9, 10 to 14, 15 to 17)<br>**Sex**: Male or female<br>**Race/Ethnicity**: Two variables 1) Yes/No Hispanic, 2) Race: White, Black, Hispanic, Asian, Indian, Native American Indian, Alaskan Native, Native Hawaiian/Other Pacific Islander, Two or More races, Other, Unknown. Race/ethnicity was coded from these as: non-Hispanic white, non-Hispanic black, Hispanic, Asian/Pacific Islander, Other/2+ races<br>**Socio Economic Status**: Used number of members in each household and household income to determine poverty status – above/below poverty line, missing
NKI-Rockland (N=309)	*American Community Survey, 2016:*<br>**Age**: Age groups (6 to 11, 12 to 17)<br>**Sex**: Male or female<br>**Race/Ethnicity**: Hispanic, Non-Hispanic White, Black, American Indian/Alaska Native, Asian Native Hawaiian/Other Pacific Islander, Other<br>**Socio Economic Status**: Above/below the poverty line, missing	**Age**: Single ages grouped into two age groups (6 to 11, 12 to 17)<br>**Sex**: Male or female<br>**Race/Ethnicity**: Two variables 1) Yes/No Hispanic, 2) Race: White, Black, Asian, American Indian or Alaskan Native, Native Hawaiian/Other Pacific Islander, Other. Race/ethnicity was coded from these as: non-Hispanic white, non-Hispanic black, Hispanic, Other/2+ races<br>**Socio Economic Status**: Used number of members in each household and household income to determine poverty status – above/below poverty line, missing
SYS (N=1004)	*Canadian 2006 Census of Population, Quebec*:<br>**Age**: Age groups (10 to 14, 15 to 19)<br>**Adult Education**: Highest level of education received by adults aged 35-54 (1. No certificate, diploma or degree; 2. High school certificate or equivalent; 3. College, CEGEP or other non-university certificate or diploma; 4. Bachelor's degree+)<br>**Income**: Household income categories (< 15,000, 15,000-24,999, 25,000-34,999, 35,000-44999, ≥45000)	**Age**: Age in months, rounded to nearest age<br>**Parental Education**: Mother and father parental education categories (Combined: 1. No certificate, diploma or degree certificate or diploma; 2. High school/trade school certificate or equivalent & university studies not completed; 3. College, CEGEP or other non-university certificate or diploma; 4. Bachelor's degree & above bachelor level; 5. Missing). Used Father's education and matched it to adult male education.<br>**Income**: Household income categories (< 15,000, 15,000-24,999, 25,000-34,999, 35,000-44999, ≥45000, missing)

Note: Data for the sample weight for HBN and NKI-Rockland were drawn from the 2016 American Community Survey. Data for the sample weight for SYS were drawn from the 2006 Canadian Census of Population.

© 2020 Margolis AE et al. *JAMA Network Open.*

eTable 3. HBN and NYC* Populations and Variables Used to Create a Sample Weight

Sociodemographic Variables for NYC Children	2016 NYC Population (N =1,340,375)		HBN (N=1283)		HBN with weight	HBN sensitivity weight - 1% trim	NVLD ONLY (N=137)	
	N	%	N	%	%	%	N	%
Weighted Variables								
**Race**								
Non-Hispanic White	335,213	25.0	607	47.3	25.0	29.7	57	41.6
Non-Hispanic Black	318,564	23.8	165	12.9	23.8	22.6	30	21.9
Hispanic	479,586	35.8	315	24.6	35.8	35.6	33	24.1
Asian/Pacific Islander	170,127	12.7	36	2.8	12.7	8.0	5	3.7
Other/ 2+ Races	36,952	2.8	160	12.5	2.8	4.1	12	8.8
**Sex**								
Male	682,251	50.9	796	62.0	50.9	53.0	89	65.0
Female	658,124	49.1	487	38.0	49.1	47.0	48	35.0
**Income**								
Below Poverty Line	384,688	28.7	56	4.4	28.7	21.3	11	8.0
Above Poverty Line	942,394	70.3	810	63.1	70.3	74.2	95	69.3
Missing income	13,060	1.0	417	32.5	1.0	4.5	31	22.6
Nested Variable								
**Age**								
6 to 9 years	392,635	29.3	543	42.3	29.3	33.8	41	29.9
10 to 14 years	465,408	34.7	539	42.0	34.7	36.1	59	43.1
15 to 19 years	482,332	36.0	201	15.7	36.0	30.1	37	27.0

Note: * NYC population pulled from the 2016 American Community Survey

© 2020 Margolis AE et al. *JAMA Network Open.*

eTable 4. NKI and Rockland* Populations and Variables Used to Create a Sample Weight

Sociodemographic Variables	Rockland (N = 59,807)		Overall NKI Sample (N = 309)		NKI with Weight	NKI trim weight - 1% trim	NVLD ONLY (n = 30)	
	N	%	N	%	%	%	N	%
Weighted Variables								
**Race**								
White	38336	64.1	171	55.3	64.1	63.5	14	46.7
Black	5024	8.4	53	17.2	8.4	8.7	6	20.0
Hispanic	11722	19.6	57	18.5	19.6	19.9	7	23.3
Other	4725	7.9	28	9.1	7.9	8.0	3	10.0
**Sex**								
Male	30502	51.0	174	56.3	51.0	50.9	15	50.0
Female	29305	49.0	135	43.7	49.0	49.1	15	50.0
**Poverty**								
Below Poverty Line	14497	24.2	20	6.5	24.2	22.9	5	16.7
Above Poverty Line	44918	75.1	219	70.9	75.1	76.2	19	63.3
Missing income	392	0.7	70	22.7	0.7	1.0	6	20.0
Nested Variable								
**Age**								
6 to 11	30981	51.8	167	54.1	51.8	52.6	25	83.3
12 to 17	28826	48.2	142	46.0	48.2	47.4	5	16.7

Note: * Rockland population pulled from the 2016 American Community Survey

© 2020 Margolis AE et al. *JAMA Network Open.*

eTable 5. SYS and Saguenay Populations and Variables Used to Create a Sample Weight

Sociodemographic Variables	Saguenay (N = 7533)		Overall SYS Sample (N = 1004)		SYS with Weight	SYS trim weight - 1% trim	NVLD (n = 29)	
	Estimated N	%	N	%	%	%	N	%
Weighted Variables								
**Father's Education**								
No certificate, diploma or degree	980	13.0	165	16.4	13.0	13.0	4	13.8
High school/trade school certificate or equivalent	3701	49.1	465	46.3	49.1	49.2	18	62.1
College, CEGEP or other non-university certificate or diploma	1396	18.5	200	19.9	18.5	18.6	3	10.3
university studies not completed	329	4.4	60	6.0	4.4	4.4	0	0.0
Bachelor's degree +	1127	15.0	114	11.4	15.0	14.9	4	13.8
**Income**								
less than 15000	391	5.2	71	7.1	5.2	5.2	3	10.3
15000-24999	608	8.1	69	6.9	8.1	8.0	1	3.5
25000-34999	727	9.7	83	8.3	9.7	9.7	1	3.5
35000-44999	827	11.0	119	11.9	11.0	11.0	4	13.8
>=45000	4970	66.0	653	65.0	66.0	66.1	20	69.0
missing	10	0.1	9	0.9	0.1	0.1	0	0.0
Nested Variable								
**Age**								
12 to 14	2688	35.7	413	41.1	35.7	35.7	7	24.1
15 to 19	4845	64.3	591	58.9	64.3	64.3	22	75.9

© 2020 Margolis AE et al. *JAMA Network Open.*

eTable 6. Inflation Rate by Specific Disorder

Sample	Diagnosis	Sample Rate	Sample Weighted Rate -1% trim	Population Rate*	Inflation Rate**
HBN					
	Autism	9.59	8.52	1.7	5.01
	ADHD	56.35	53.65	8.7	6.17
	SLD	18.71	17.55	4.8	3.66
	Anxiety	26.81	30.29	31.9	0.95
	Depression	9.9	13.07	13.3	0.98
NKI					
	ADHD	22.65	26.52	8.7	3.05
	Anxiety	13.27	16.26	31.9	0.51
	Depression	3.24	4.01	13.3	0.30

*Population rates for depression, anxiety, and ADHD were obtained from the NIH website (https://www.nimh.nih.gov/health/statistics/index.shtml), rates of Specific Learning Disorder from the National Center for Education Statistics website (https://nces.ed.gov/programs/coe/indicator_cgg.asp), and rates of Autism Spectrum Disorder from the CDC website (https://www.cdc.gov/ncbddd/autism/addm.html); see Supplemental Methods for more details.

**Inflation rate = Sample weighted rate / Population rate.

© 2020 Margolis AE et al. *JAMA Network Open.*

eTable 7. Prevalence of NVLD in HBN Without Excluding ASSQ >19

Sample	% meet criteria for NVLD			Adjusted* rate of NVLD among population
	Unweighted	Weighted	Sensitivity weighted - 1% trim (95% CI)	
HBN	9.19	17.05	15.13 (10.89-19.37)	2.45 (1.76-3.14)

Note. HBN sample including all records (N=1491) with complete demographic and neuropsychological data.

eMethods. Supplemental Methods

Population rates for known disorders

Rates were selected from published prevalence reports using data obtained through diagnostic interview data or child performance measures, rather than parent report of a diagnosis via interview, whenever possible.

Rate of Attention Deficit Hyperactivity Disorder (ADHD) was obtained from the NIMH website (https://www.nimh.nih.gov/health/statistics/attention-deficit-hyperactivity-disorder-adhd.shtml). Data from National Comorbidity Survey–Adolescent Supplement (NCS-A) documents the lifetime prevalence of ADHD among U.S. adolescents aged 13 to 18 years as 8.7 percent.

Rate of Specific Learning Disability (SLD) was calculated based on information obtained from the National Center for Education Statistics website (https://nces.ed.gov/programs/coe/indicator_cgg.asp). The site reported that in 2017-18, 7 million children (14% of all public school students) received special education services, and of these 34 percent had SLD. Accordingly, 2,380,000 children (34% of those receiving special education services) of the total population of 50 million public school children had an SLD classification, yielding a prevalence of 4.8 percent.

Rate of Autism Spectrum Disorder (ASD) was obtained from the Center for Disease Control (CDC) website (https://www.cdc.gov/ncbddd/autism/addm.html). Data from the Autism and Developmental Disabilities Monitoring (ADDM) Network documents that 1 in 59 8-year old children met criteria for ASD.

Rate of anxiety was obtained from the NIMH website (https://www.nimh.nih.gov/health/statistics/any-anxiety-disorder.shtml). Data from National Comorbidity Survey Adolescent Supplement (NCS-A), documents that 31.9 percent of adolescents had any anxiety disorder.

Rate of major depression was obtained from the NIMH website (https://www.nimh.nih.gov/health/statistics/major-depression.shtml). Data from Substance Abuse and Mental Health Services Administration (SAMHSA) documents

that in 2017, 13.3 percent of adolescents had had at least one major depressive episode.

Coding

R code used for sample weighting procedures is available upon request.

eAppendix. Supplemental Results

Demographic differences in sample frequencies compared with the underlying target populations

Relative to the ACS sample, the unweighted HBN sample had more participants age 6-9 and age 10-14 (42 vs 29%; 42 vs 35% respectively) and fewer age 15-17 (16 vs 36%), more Non-Hispanic White participants (47 vs 25%) and fewer Non-Hispanic Black, Hispanic and Asian/Pacific Islander participants (13 vs 24%; 25 vs 36%; 3 vs 13%, respectively), more males and fewer females (62 vs 51% males), and fewer below the poverty line (4 vs 29%), roughly the same above the poverty line (63 vs 70%) and many more with missing income data (33 vs 1%). All information can be found in Table S3.

Relative to the ACS sample, the unweighted NKI sample had more participants age 6-11 and fewer participants age 11-17 (54 vs 52%; 46 vs 48%, respectively), fewer White (55 vs. 64%) and Hispanic descent (18 vs 20%) and a greater proportion of Black (17 vs. 8%) and other race participants (9 vs. 8%), more males (56 vs 51%), and fewer individuals living below and above the poverty line (6 vs 24%; 71 vs 75%, respectively) and more with missing income data (23 vs 0.7%). All information can be found in Table S4.

Relative to the census, the SYS sample had more participants age 12-14 and fewer participants age 15-19 (41 vs 36%; 59 vs 64%, respectively), fewer participants with more than a Bachelor's degree and a high school certificate or equivalent (11 vs 15%; 46 vs 49%, respectively), roughly the same among those with College, CEGEP or other non-university certificate or diploma (20 vs 19%), whereas more participants had no certificate, diploma or degree (16 vs 13%), and roughly equal numbers of participants across household annual income categories: higher then 45,000 (65 vs 66%), between 35-44,999 (12 vs 11%), between 25-34,999 (8 vs 10%), between 15-24,999 (7 vs 8%), and less than 15,000 (7 vs 5%). All information can be found in Table S5.

Demographic characteristics of children who met criteria for NVLD

The distribution of NVLD (N = 137) in HBN across demographic groups is similar to the overall breakdown of HBN (Table S3). The highest proportion of participants with NVLD were non-Hispanic White (42%) followed by Hispanic (24%), then Non-Hispanic Black (22%), and male participants made up 65% of those with NVLD. Participants above the poverty line made up the largest proportion of NVLD (69%) followed by those missing income (24%). Last, the age group with the most participants with NVLD was the 10-14 year olds (43%), followed by the 6-9 year olds (30%).

The distribution of NVLD in NKI (N = 30) across demographic groups is similar to the overall breakdown of NKI (Table S4). The highest proportion of participants with NVLD were White (47%) followed by Hispanic (23%), then Non-Hispanic Black (20%), and male participants made up 50% of those with NVLD. Participants above the poverty line made up the largest proportion of NVLD (63%) followed by those missing income (20%). Last, the age group with the most participants with NVLD was the 6-11 year-olds (83%).

The distribution of NVLD in SYS (N = 29) across father's education and income groups differs slightly from the overall SYS sample (Table S5). The highest proportion of participants with NVLD had a father who completed high school/trade school certificate (62%) followed by no diploma/degree or more than a bachelor's degree (both 14%). Participants with a household income of more than $45,000 made up the largest proportion (69%), followed by $35,000-44,999 (14%), and less than $15,000 (10%). No participants with NVLD were missing data on income. Last, the age group with the most participants with NVLD was the 15-19 year-olds (76%).

© 2020 Margolis AE et al. *JAMA Network Open.*
